# Effects of pharmacist intervention in Vancomycin treatment for patients with bacteremia due to Methicillin-resistant *Staphylococcus aureus*

**DOI:** 10.1371/journal.pone.0203453

**Published:** 2018-09-06

**Authors:** Atsushi Komoto, Takayoshi Maiguma, Daisuke Teshima, Tetsuhiro Sugiyama, Yuto Haruki

**Affiliations:** 1 Graduate School of Pharmacy, Shujitsu University, Okayama, Okayama, Japan; 2 Department of Pharmacy, Tsuyama Chuo Hospital, Tsuyama, Okayama, Japan; Kent State University, UNITED STATES

## Abstract

**Objective:**

We conducted a retrospective study based on composite endpoints for treatment failure to evaluate the effect of pharmacist-led VCM initial dose planning for Methicillin-resistant *Staphylococcus aureus* (MRSA) bacteremia patients.

**Methods:**

A retrospective cohort study was performed between pharmacist intervention and non-intervention groups. In this study, four types of failure were defined as the composite endpoint. When any one of the following failures occurred: 1) Death within 30 days from the start of VCM therapy, 2) Positive blood culture 7 days after the start of VCM therapy, 3) Change of VCM to another anti-MRSA agent, and 4) Development of nephrotoxicity, we considered that VCM treatment had failed. Survival time analysis was conducted with the Kaplan-Meier method and Cox’s proportional hazard model that included seven predefined parameters: pharmacist intervention, age, sex, weight, baseline VCM trough concentration, Charlson Comorbidity Index (CCI), and Pitt Bacteremia score (PBS). The effect of pharmacist intervention was studied as the survival probability estimated from the period of time from the start of VCM administration to the earliest failure.

**Results:**

The survival rate at 30 days after starting VCM therapy, at the end of follow-up, was 53.1 and 82.1% in the non-intervention and intervention groups, respectively. A significant survival time prolongation was noted in the intervention group (p = 0.011, log rank test). Among the seven parameters, only pharmacist intervention was significantly different and its hazard ratio was 0.26 (p = 0.014). The survival probability of the intervention group was higher than that of the non-intervention group for the time to each failure. In subgroup analyses, a significant difference was noted in male patients between the intervention and non-intervention groups (p = 0.005). Age was categorized into those under and over 65 years old. For those over 65 years old, a significant difference was shown between the groups (p = 0.018).

**Conclusion:**

To our knowledge, this is the first study to evaluate the failure of VCM treatment based on the composite endpoint. Pharmacist intervention through the initial VCM dose planning could maintain a balance between the efficacy and safety of VCM treatment and might avoid treatment failure for patients with MRSA bacteremia. Further investigations with large sample sizes are required to confirm our findings.

## Introduction

Methicillin-resistant *Staphylococcus aureus* (MRSA) bacteremia is a severe nosocomial infection that has been reported to be associated with a longer hospital stay, higher mortality, and increased costs [1–3]. Vancomycin (VCM), classified as a glycopeptide antibiotic, has been used for over decades to treat MRSA infections. Although newer anti-MRSA antibiotics such as Linezolid and Daptomycin have recently become available [4], VCM still plays an important clinical role in the treatment of invasive MRSA infections. Previous studies on the pharmacokinetics and pharmacodynamics (PK/PD) of VCM showed that a ratio of the area under the curve to the minimum inhibitory concentration (AUC/MIC) of ≥400 is optimal for clinical effectiveness [5]. A VCM serum trough concentration of 15–20 μg/mL is a surrogate marker to attain the target AUC/MIC ratio when the MIC is 1 μg/mL [6]. The 2009 consensus guidelines published by the American Society of Health—System Pharmacists, the Infectious Diseases Society of America, and the Society of Infectious Diseases Pharmacists recommended a VCM trough level of 15–20 μg/mL [7]. On the other hand, nephrotoxicity, a major side effect of VCM therapy, was significantly correlated with high trough concentrations of VCM (>20 μg/mL) [8]. Lodise et al. reported that larger VCM doses (at least four grams per day) based on initial high-dose planning (15–20 μg/mL) led to a higher incidence of nephrotoxicity than a standard dose [9]. Therefore, in Japan, the practice guidelines for VCM TDM published by the Japanese Society of Chemotherapy and the Japanese Society of Therapeutic Drug Monitoring recommend an initial target trough concentration of VCM of 10–20 μg/mL [10]. Some studies have demonstrated that VCM dose planning by pharmacists could lead to a higher percentage of patients with an optimal VCM trough concentration [11–13]. However, it remains unclear whether pharmacist intervention is associated with a better clinical outcome and improved safety in patients with complicated MRSA infection resulting from the appropriate VCM trough concentration. Therefore, we conducted a retrospective study for the purpose of evaluating the effect of pharmacist-led VCM initial dose planning for MRSA bacteremia patients.

## Materials and methods

### Study design and pharmacist intervention

**Fig 1:**
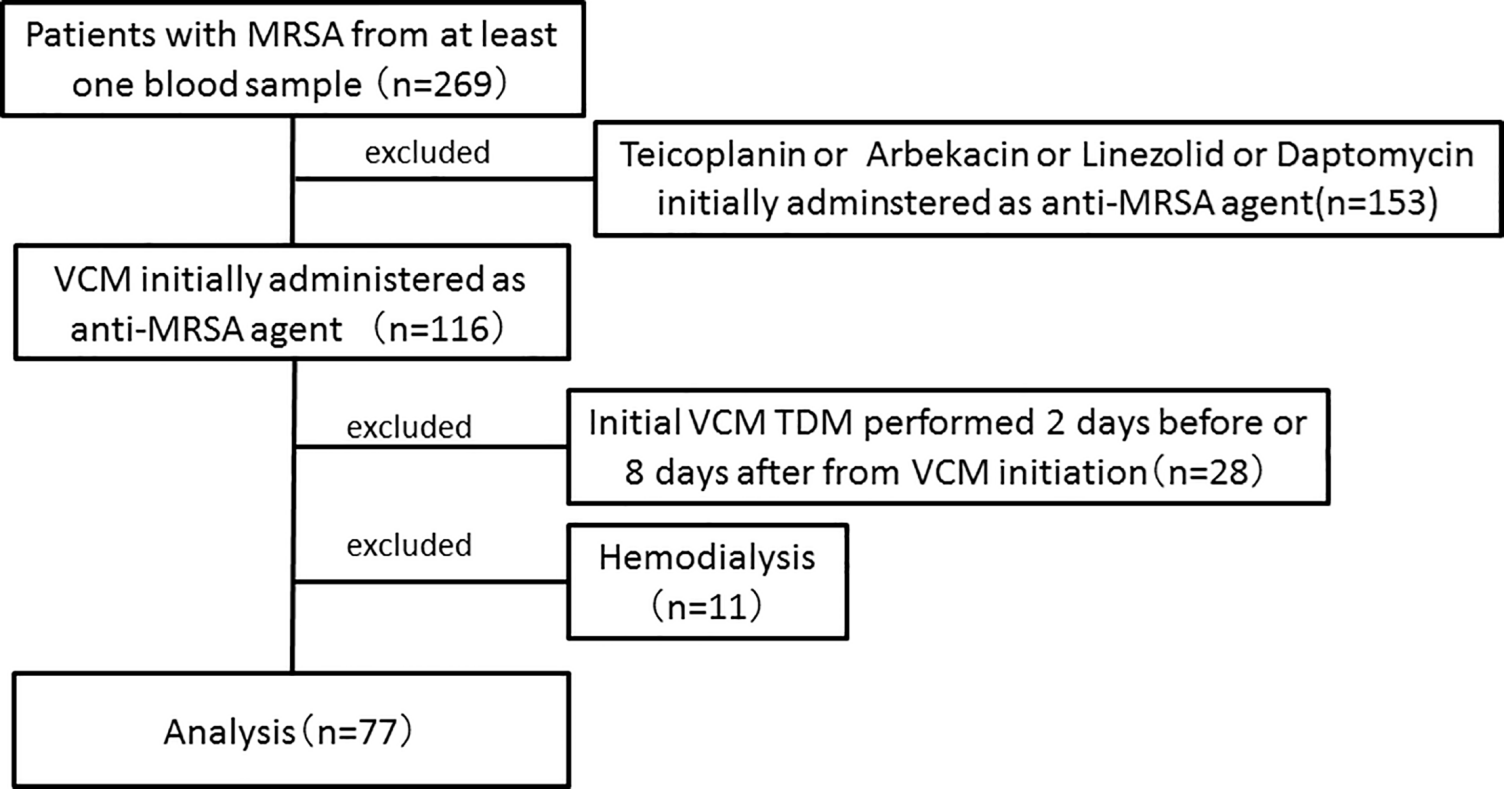
Study design.

A retrospective cohort study was performed at Tsuyama Chuo Hospital, a 535-bed acute community hospital in Japan from January 2005 to May 2016. Of 269 patients in whom MRSA was detected from one or more sets of blood culture during the specified period, 116 patients treated with VCM as the first anti-MRSA agent were extracted. Those treated first with other anti-MRSA agents: teicoplanin, arbekacin, linezolid, and daptomycin, were excluded (153 patients). Since they were treated with anti-MRSA agents, all patients were judged as having infectious disease and the detection was not contamination. Patients requiring hemodialysis (11 cases) were excluded. In addition, the first TDM performed within 2 days after initiation of administration was excluded because the blood VCM level may not have reached a steady state, and the first TDM performed 8 or more days after initiation of administration was excluded because a long time has passed after administration and the clinical effect of VCM at the blood VCM level may not be accurately judged. Finally, 77 patients were analyzed and evaluated (Fig 1). All the analyzed patients were over 18-year-old.

Although the pharmacists monitored the blood level after the dosing of VCM, from January 2005 to May 2012, the pharmacists were not involved in the initial dose planning of VCM, because of the doctors’ initiative. Therefore, we defined the patients in this period of time as a non-intervention group of pharmacists.

**Fig 2:**
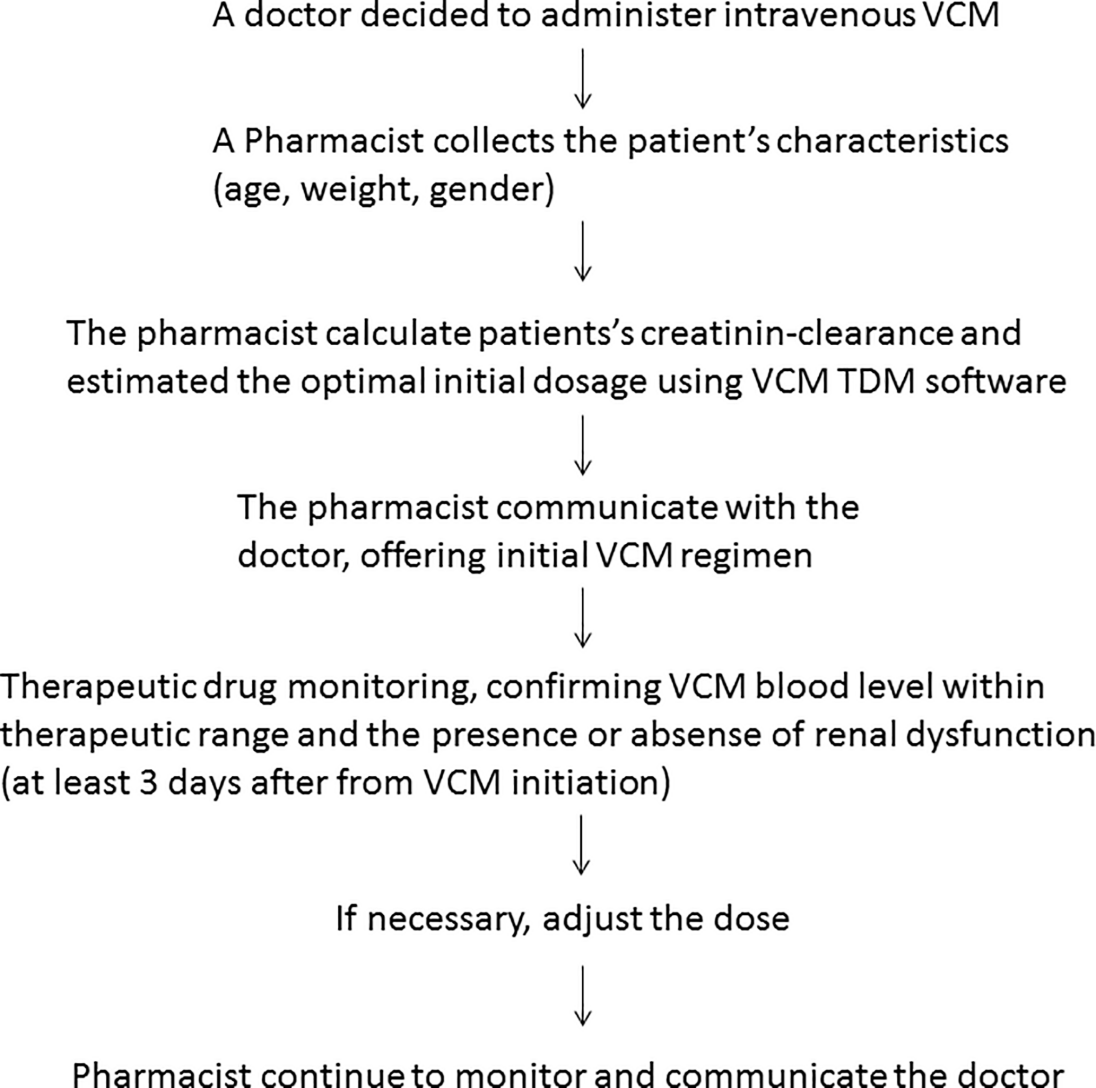
Flow chart of pharmacist intervention of vancomyacin treatment.

On the other hand, from June 2012 to May 2016, the pharmacists intervened in the initial dose planning using VCM TDM software (SHIONOGIVCM-TDM S-edition ver. 2009, Shionogi Inc., Japan or Vancomycin MEEK TDM analysis software Ver. 3.0, Meiji Seika Pharma Inc., Japan) [14, 15]. We defined those in this period as the intervention group of pharmacists. A flowchart of the pharmacist intervention method is shown in Fig 2. In this period, when a doctor prescribed intravenous VCM, a pharmacist intervened in all the cases to support VCM initial dose planning. The pharmacist intervention has been performed 24–7. Each pharmacist collected information on the diagnosis and the patient’s information such as age, sex, body-weight, and especially serum creatinine (Scr). Based on the collected information, the pharmacist calculate their creatinin-clearance and estimated the optimal initial dosage using a bayesian forecasting technique and presented the regimen to the doctors. Then the doctor prescribed VCM according to the pharmacist’s recommendation.

The target VCM trough concentration was set at 10–20 μg/mL based on Japanese TDM guidelines [8]. To avoid overdosing in the initial VCM dose planning, the Scr value was handled as 0.6 in cases below 0.6 Scr [16]. We basically set the initial VCM TDM day as 3 days after the VCM initiation.

Since pharmacists intervened after the first TDM throughout the study period, the only difference between the 2 groups was the presence or absence of the initial dose planning prepared by pharmacists. The date of the first TDM in the intervention group and the dates of the second and later TDM in the 2 groups were proposed by pharmacists, but the date of checking the renal function and the schedule of blood culture re-test were not specified and these were performed at the physician’s judgement.

A retrospective chart review was performed for patients’ clinical characterstics such as age, sex, body weight, comorbidity, and its severity. The patients’ comorbid conditions were evaluated by the Charlson Comorbidity Index (CCI) [17], and the severity of bacteremia was evaluated by the Pitt Bacteremia Score (PBS) [18]. Intergroup comparison was conducted between pharmacist intervention and non—intervention groups.

The present study was approved by both the Ethics Committee of Tsuyama Chuo Hospital (No. 171) and Shujitsu University (No.90). Information on all patients was from the Tsuyama Chuo Hospital database. All data remained anonymous so that individuals could not be identified from the database.

### Microbial analysis and laboratory data

A signal blood culture system (Oxoid USA Inc., Columbia, MD, USA) was used prior to 2007, and a BacT/Alert 3D system (Sysmex bioMerieux, Tokyo, Japan) was used thereafter. Bacterial identification and antibiotic susceptibility testing were conducted using the Microscan Walkaway 40 SI system (Siemens Healthcare Diagnostics, Tokyo, Japan). Serum VCM concentrations were measured by the TDX FLX analyzer (Abbott Laboratories, Lake Bluff, IL, USA) from January 2005 to May 2010, and the Viva-E^®^ System (Siemens, Newark, DE, USA) was used subsequently.

### Endpoints

In this study, four types of failure were defined, and when they occurred, the effect of pharmacist intervention was studied as the survival probability estimated from the period of time from the start of VCM administration. If any treatment failure of the following four types was observed, we decided that the VCM treatment had failed: 1) Death within 30 days from the start of VCM therapy, 2) Positive blood culture 7 days after the start of VCM therapy, 3) Change of VCM to another anti-MRSA agent, and 4) Development of nephrotoxicity. The reason for switching from VCM to another anti-MRSA agent was judged based on the medical record written by the attending physician. Switching considered due to VCM-induced allergy was excluded, and switching due to renal dysfunction was also excluded because it was defined in 4), and only switching due to insufficiency of the treatment effect of VCM was included. Nephrotoxicity was defined as an increase in serum creatinine (Scr) of more than 0.5 mg/dL or a 50% increase from the baseline for 30 days from the date of the first VCM therapy [7]. The composite endpoint was adopted in this study. We defined the time to failure of VCM therapy as the period of time from the start of VCM administration to failure involving the four events mentioned above. Regarding the primary endpoint, development of any one of the events defined in 1)-4) was regarded as failure of VCM treatment. The secondary endpoint was defined as the period of time from the start of VCM administration to each type of failure.

### Statistical analysis

Baseline characteristics were compared between the intervention and non-intervention groups. Continuous variables were compared by Student’s t-test, and categorical variables by the chi-squared test. Fisher’s exact test was performed when the expected frequencies were below five. Survival time analysis involved the Kaplan-Meier method and Cox’s proportional hazard model that included seven predefined parameters: pharmacist intervention, age, sex, weight, baseline VCM trough concentration, CCI and PBS. All reported P-values are two- sided, and confidence intervals were at the 95% level. Data were analyzed with the use of JMP^®^ 13 (SAS Institute Inc., Cary, NC, USA).

## Results

### Baseline characteristics of patients

**Fig 3:**
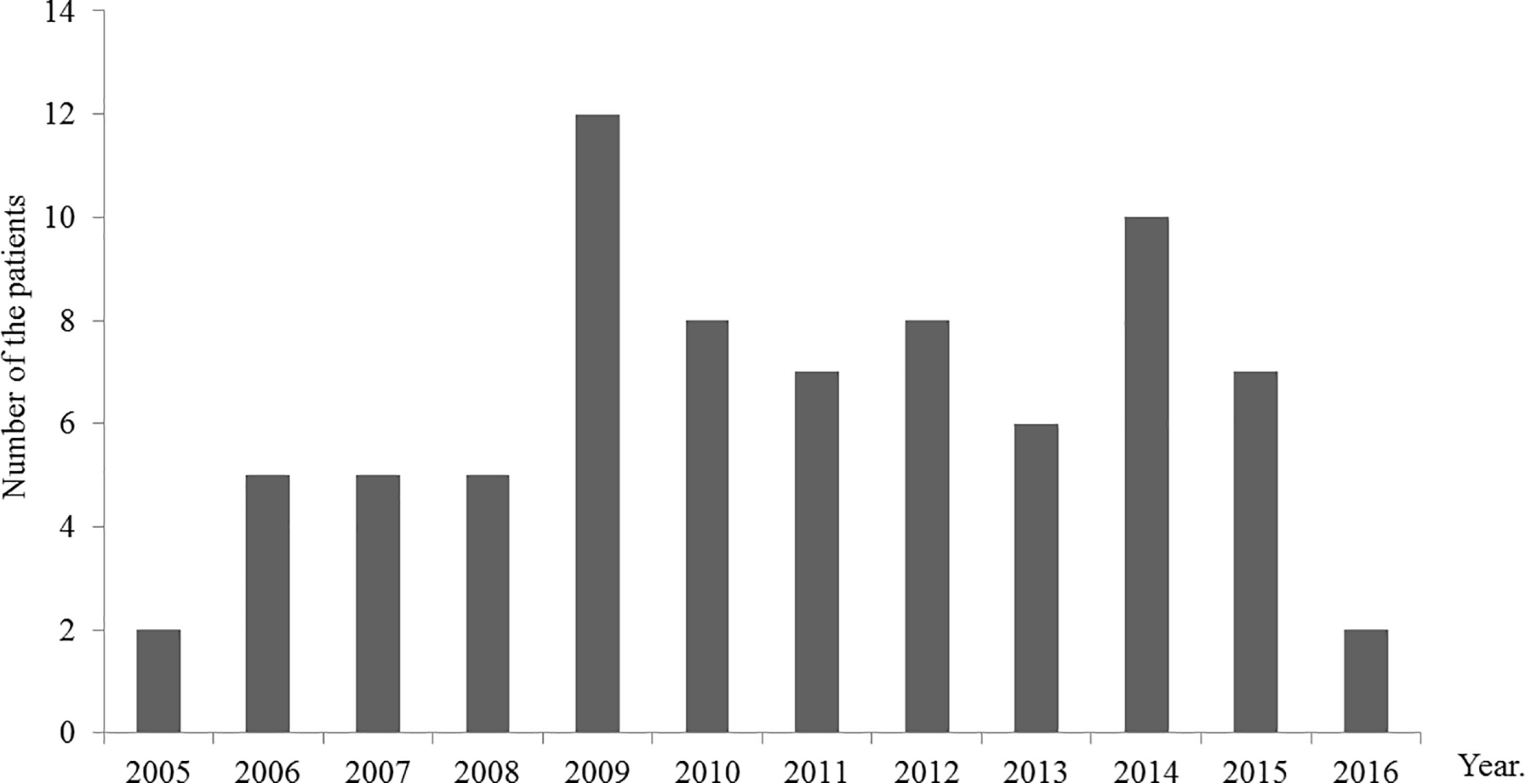
Distribution of the number of analyzed patients per year in this study.

Baseline characteristics of patients are summarized in Table 1. PBS, which evaluated the severity of bacteremia, was 2 (interquartile range: 1–4) for the non-intervention group and 1 (0–3) for the intervention group, being significantly lower in the intervention group (p = 0.019). Mean trough level was 17.6 ± 10.20 μg/mL for non-intervention group and 11.2 ± 3.25 μg/mL for intervention group, being significant lower in the intervention group (p = 0.002). Percent that target on first trough was 40.8% for non-intervention group and 64.3% for intervention group, being significant higher in the intervention group (p = 0.048). Mean mg/kg dose was 29.1 ± 12.5 for non-intervention group and 21.1 ± 9.41 for intervention group, being significant lower in the intervention group (p = 0.004). The other characteristics such as age, sex, weight, CCI and VCM MIC had no significant difference between the groups. The number of patients included in analysis in each year during the specified period is shown in Fig 3. Mean value was 6.4, and range was 2–12.

**Table 1:** Baseline characteristics of patients.

	Non-intervention group	Intervention group	p value
**Number of patients**	49	28	-
**Age**^a^	74.7 ± 1.72	79.8 ± 2.22	0.074
**Sex (male %)**	31 (68.9%)	13 (48.2%)	0.081
**Weight**^a^ **(kg)**	50.9 ± 1.48	51.5 ± 1.91	0.798
CCI*^b^	2 [0, 3]	3 [2, 5]	0.101
PBS*^b^	2 [1, 4]	1 [0, 3]	0.019
**Trough Levels**^a^	17.6 ± 10.2	11.2 ± 3.25	0.002
**Percent that hit target on first trough**	40.8 (20/49)	64.3 (18/28)	0.048
**mg/kg dose**^a^	29.1 ± 12.5	21.1 ± 9.41	0.004
VCM MIC* **(<2mg/liter %)**	44 (89.8%)	26 (92.9%)	0.653

*CCI: Charlson Comorbidity Index, PBS: Pitt Bacteremia score, VCM MIC: Vancomycin MIC.

^a^ Data shown as mean ±standard deviation.

^b^ Data shown as median [interquartile range].

The initial dose plans were prepared by pharmacists in all VCM-treated patients in the intervention group and the acceptance rate was 100%.

### Effect of pharmacist intervention on the primary endpoint based on Kaplan-Meier estimation

**Fig 4:**
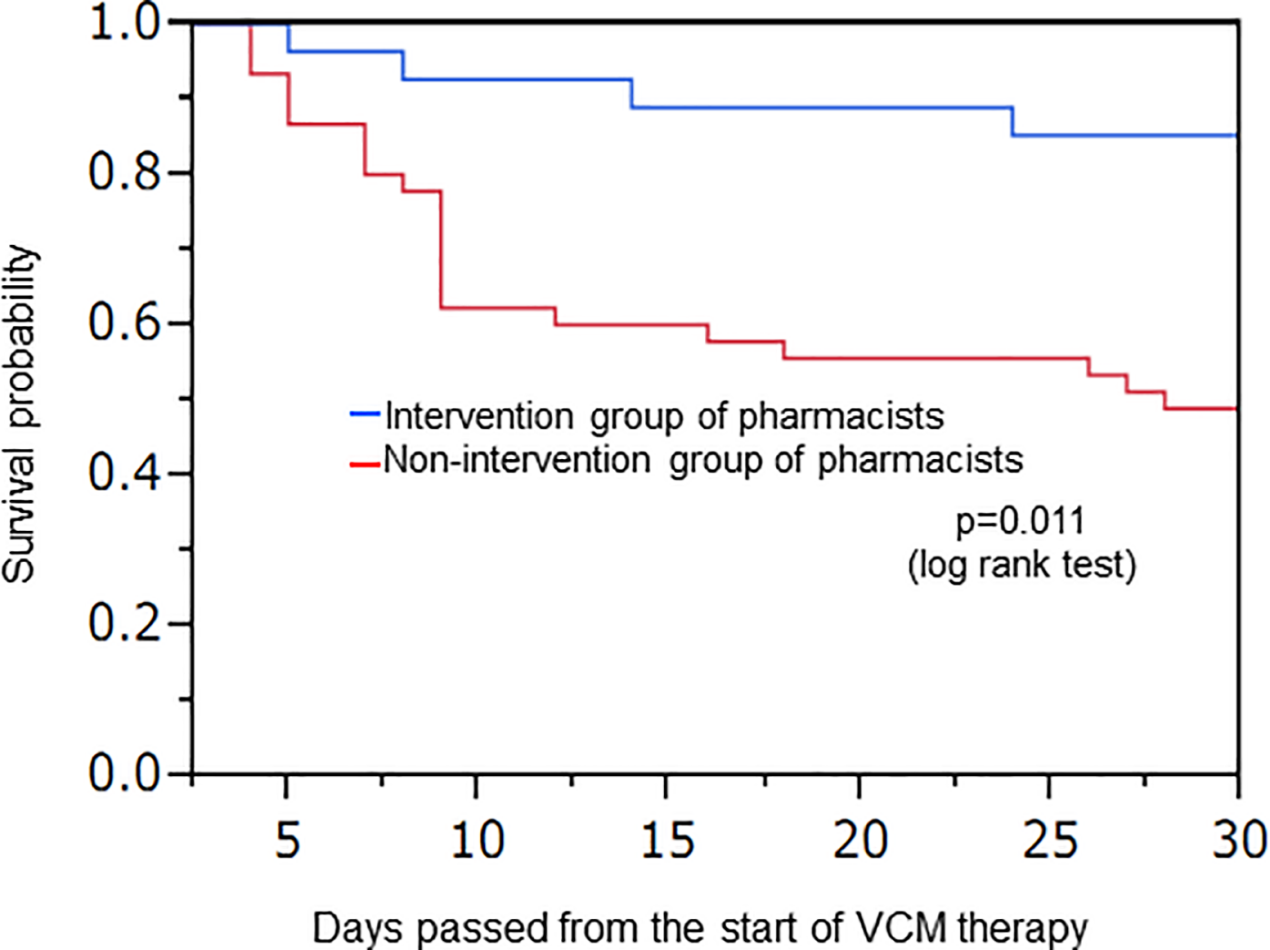
Effect of intervention by a pharmacist based on the Kaplan-Meier estimate of survival curves.

The Kaplan-Meier estimate of survival curves in VCM therapy is shown in Fig 4. The survival rate 30 days after starting VCM therapy, at the end of follow-up, was 53.1 and 82.1% for non-intervention and intervention groups, respectively. A significant survival time prolongation was noted in the pharmacist intervention group (p = 0.011, log rank test).

### Factors involved in the period of time to failure of VCM therapy

The results of regression analysis using the proportional hazard model for the period of time to failure of VCM therapy are shown in Table 2. Among the seven parameters, only pharmacist intervention was significantly different and its hazard ratio was 0.26 (p = 0.014). Because PBS, which was significantly different in Table 1, showed no significant difference in Table 2, PBS had no effect on VCM treatment failure.

**Table 2:** Regression analysis using the proportional hazard model for the time to failure of VCM therapy.

	Hazard ratio	95% CI	p value
**Pharmacist intervention**	0.26	(0.077, 0.770)	0.014
**Age**	0.99	(0.958, 1.031)	0.675
**Sex (male)**	1.36	(0.575, 3.442)	0.490
**Weight (kg)**	0.97	(0.927, 1.008)	0.115
**VCM trough (μg/dL)**	0.99	(0.950, 1.033)	0.688
**CCI**	1.07	(0.854, 1.313)	0.556
**PBS**	0.97	(0.769, 1.202)	0.786

### Kaplan-Meier plots for the period of time to each type of failure

**Fig 5:**
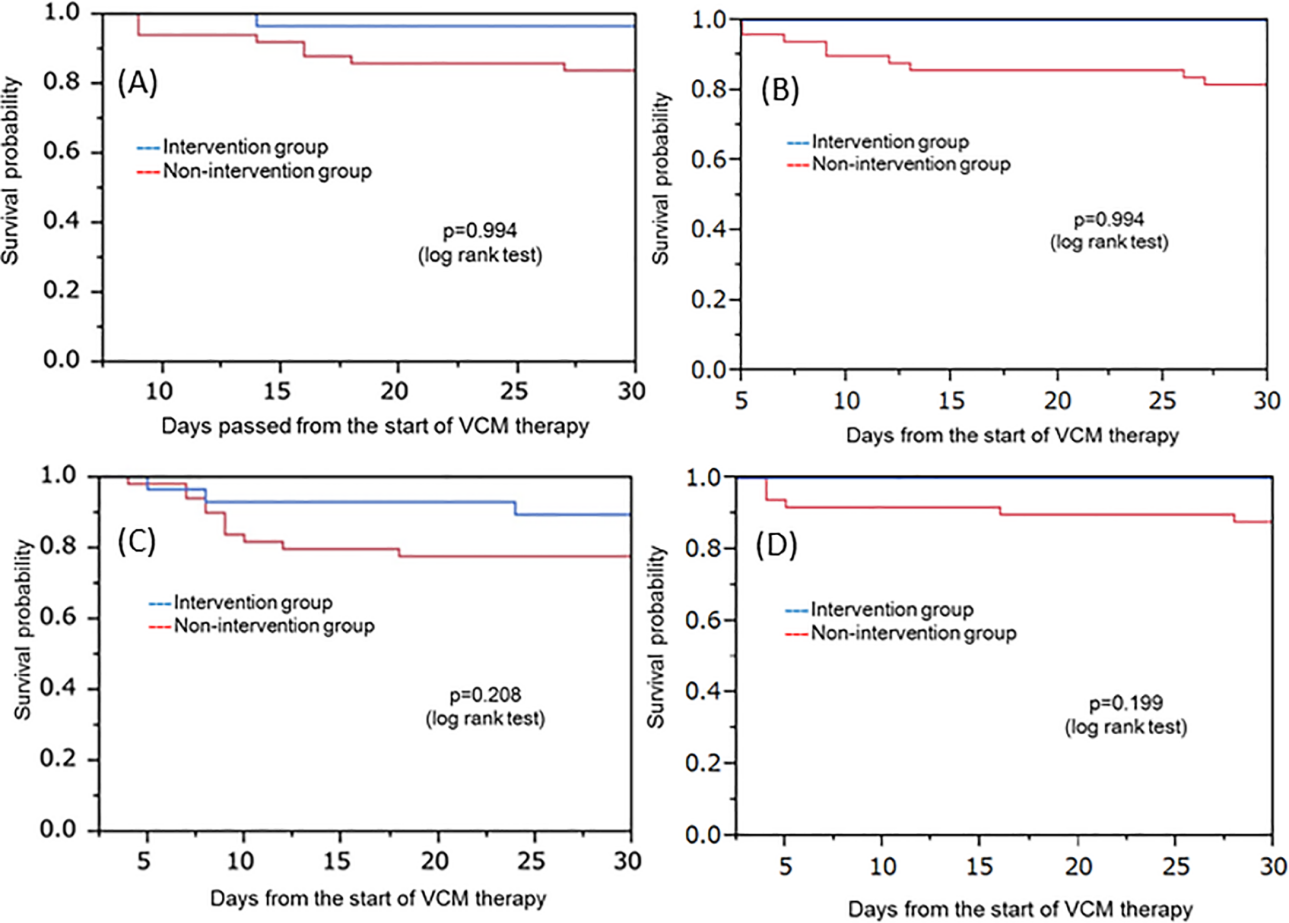
Kaplan-Meier plots for the period of time to each type of failure. (A) Kaplan-Meier plot for the time to death. (B) Kaplan-Meier plot for the time to the occurrence of positive blood culture 7 days after the start of VCM therapy. (C) Change of VCM to another anti-MRSA agent. (D) Kaplan-Meier plot for the time to the development of nephrotoxicity.

The Kaplan-Meier estimate of survival curves based on the secondary endpoint are shown to confirm the appropriateness of the Kaplan-Meier estimation based on the primary endpoint in Fig 5. The survival probability of the intervention group was higher than that of the non-intervention group for the time to each failure.

### Subgroup analyses

Subgroup analyses were also conducted for the time to failure of VCM therapy for age, sex, VCM trough level, CCI, and PBS. A significant difference was observed in the survival probability for sex and age.

**Fig 6:**
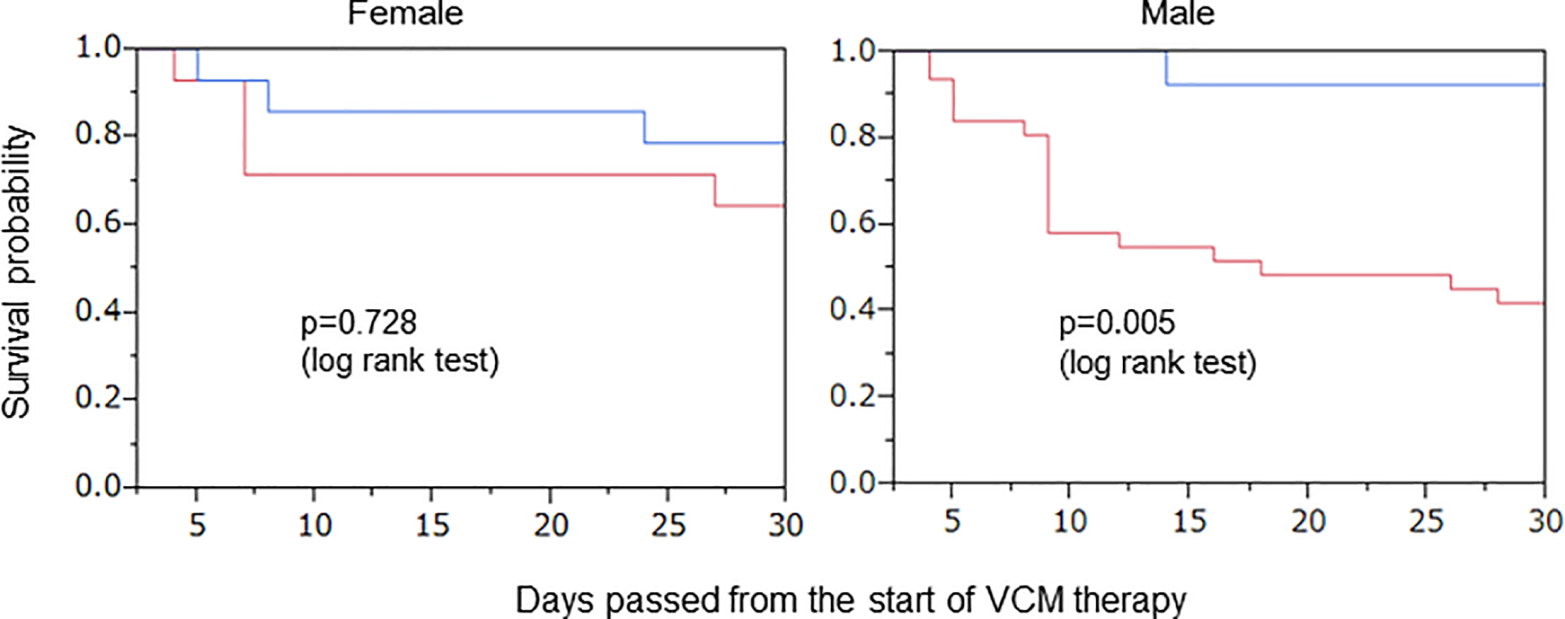
Kaplan-Meier plot for the time to failure of VCM therapy by sex. **—**Intervention group of pharmacists.—Non-intervention group of pharmacists.

**Fig 7:**
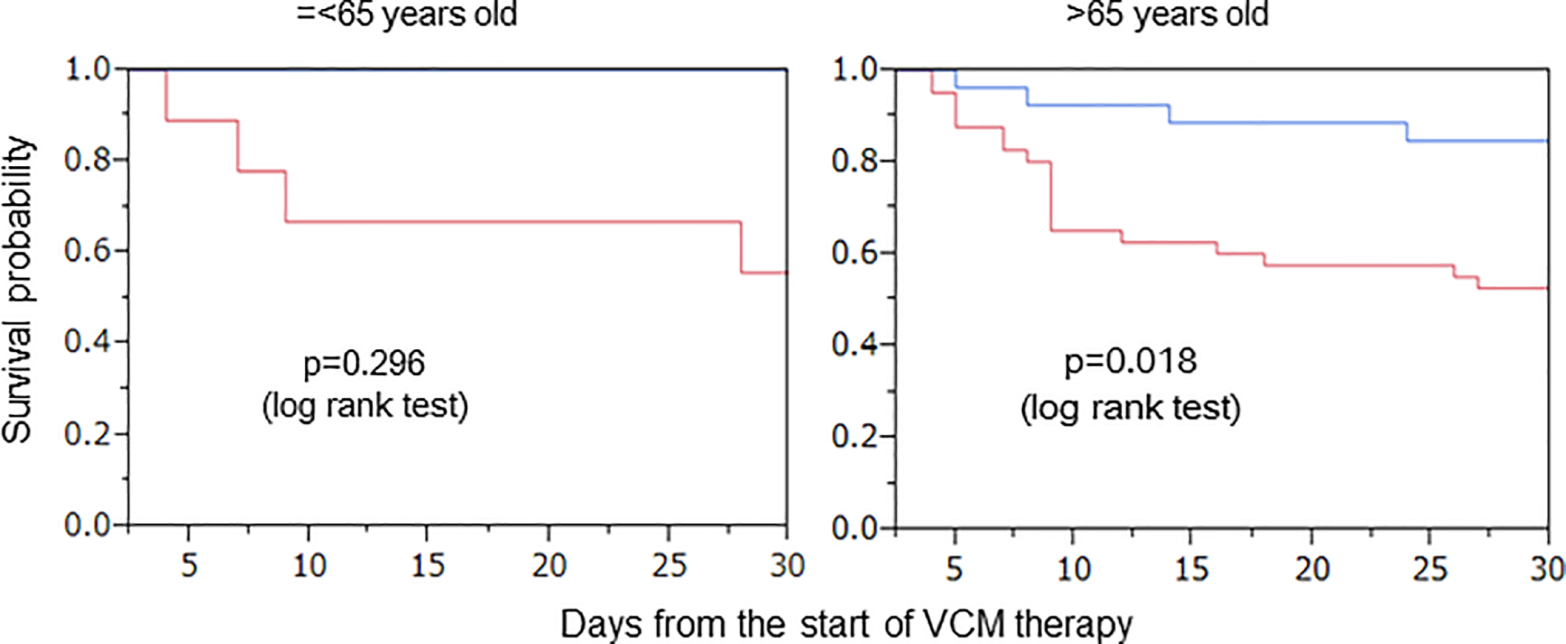
Kaplan-Meier plot for the time to failure of VCM therapy by age. **—**Intervention group of pharmacists.—Non-intervention group of pharmacists.

The results are shown in Figs 6 and 7. For male patients, a significant difference was shown between intervention and non-intervention groups (p = 0.005). However, it was not shown in females (p = 0.728). Age was categorized into those under and over 65 years old. For patients over 65 years old, a significant difference was shown between groups (p = 0.018). On the other hand, it was not shown in those under 65 years old (p = 0.296).

## Discussion

This is the first study to evaluate the failure of VCM treatment by integrating four types of endpoint. Some studies found that the effectiveness of VCM was correlated with the AUC of VCM [5, 19]. It is important to achive the therapeutic trough concentration range of VCM more rapidly and consistently because VCM needs to contact bacteria for a long time to have effects [20]. Miyazaki et al. demonstrated that the bactericidal activity of VCM was closely associated with 30-day mortality in patients with MRSA bacteremia initially administered VCM [21].

In addition, Blot et al. reported that critically ill patients needed to achieve sufficient VCM exposure more rapidly [22]. Cardile et al. reported that the time to discharge and duration of VCM treatment may be shortened by achieving the effective blood level of VCM within 5 days after initiation of administration [23]. In this study, by reaching the therapeutic concentration of VCM through pharmacist intervention, VCM bactericidal activity could be accelerated, leading to the effectiveness of VCM. A previous study reported that an adequate initial treatment reduced the mortality risk associated with MRSA bacteremia [24]. The antimicrobial stewardship program for VCM-treated patients led to a decrease in the mortality rate in a study [25]. Therefore, pharmacist intervention is necessary for appropriate initial VCM treatment (Table 2).

We previously reported that the incidence of renal dysfunction could be decreased by pharmacist intervention through the initial dose planning [26]. It has also been reported that the incidence of renal dysfunction increases in correlation with elevation of the initial trough level [27]. The mean blood level after the initial administration was lower in the intervention than non-intervention group (11.2 μg/mL vs. 17.6 μg/mL, p = 0.002), suggesting that prevention of a high level through the initial dose planning by pharmacists led to the decrease in the incidence of renal dysfunction.

In this study, we set the primary endpoint as the composite endpoint, which consisted of three parameters of effectiveness and a parameter of safety. It has been reported that the incidence of renal impairment increased in VCM treatment when the blood VCM level increased [8, 9, 28], and a low blood VCM level served as a risk of inducing resistant bacteria [29] in addition to insufficiency of the therapeutic effect [19]. Thus, both high and low blood VCM levels should be avoided. Since continuation of VCM treatment is difficult under either condition, it is appropriate to consider both conditions VCM treatment failure. Thus, it is important to maintain a balance between the efficacy and safety at the same time during VCM treatment, and intervention by pharmacists through the initial VCM dose planning may maintain this balance. The introduction of a composite endpoint might be reasonable in this study. Although several studies on the effect of pharmacist intervention for VCM treatment have been reported, improvement of the effective level achievement rate was mainly investigated [11–13] and no study on the efficacy and safety, such as significant decreases in the mortality rate and incidence of renal impairment, has been reported. We set a composite endpoint to these outcomes of the efficacy and safety, considering that pharmacist intervention leads to prevention of VCM treatment failure through preventing one of these outcomes. VCM treatment failure based on the composite endpoint was significantly different between the pharmacist intervention and non-intervention groups in this study. The pharmacist intervention might lead to not only improvement of VCM efficacy but also prevention of the development of nephrotoxicity. Pharmacists intervened after the first TDM even in the non-intervention group. Therefore, the only difference between the 2 groups was the presence or absence of the initial dose planning by pharmacists, suggesting that VCM treatment failure could be prevented by preparation of the initial dose plan by pharmacists (Fig 2).

Furthermore, we conducted a subgroup analysis for sex, age, CCI, and PBS to confirm the therapeutic effect (Table 2).

The survival probability in male patients was significantly different on comparing pharmacist intervention with non-intervention groups (Fig 6). On the contrary, the survival probability in female patients showed no significant difference between the two groups. It was shown that a female sex was one of the risk factors associated with 30-day mortality in meta-analysis of patients with MRSA bacteremia [30].

Therefore, it might be difficult to improve the survival probability of females. Additionally, the estimated creatinine clearance of males was primarily lower than that of females. In this study, the pharmacists performed initial dose planning depending on each patient’s renal function. Thereby, the VCM blood concentration could enter the therapeutic concentration range more rapidly, and the development of nephrotoxicity could be prevented. Our results are similar to those reported previously [31]. On the other hand, in the pharmacist non-intervention group, unified dose planning was conducted by physicians without depending on each patient’s condition. Since the body weight of males is relatively heavier than that of females, VCM dosage of males was probably insufficient to achieve an appropriate VCM blood concentration. Consequently, it was considered that the survival probability would decrease (Fig 6).

On comparing the pharmacist intervention with non-intervention group, the survival probability showed a significant difference in patients over 65 years old (Fig 7). However, there was no significant difference in those younger than 65 years old. Several investigators previously reported that there are some independent predictors of VCM failure, such as prior VCM exposure, an older age, and certain underlying disease states [32, 33]. Our study was consistent with the reports mentioned above. When elderly patients, who often develop renal dysfunction, are administered VCM, careful VCM dose planning would be necessary. Pharmacists should consider both the efficacy and safety of VCM when they plan the initial VCM dose.

In addition, although the survival probability was also studied for CCI and PBS between the pharmacist intervention and non-intervention groups, a significant difference was neither observed in CCI nor PBS between the two groups (Table 2).

This single-centered, retrospective study has several limitations. Since the pharmacist intervention group was biased to the latter of the study period, the development of medical techniques, devices, or instruments might have affected our results. Our hospital-initiated intervention by the infection control team for blood culture-positive patients in 2011. Reduction of the morality rate by execution of the antimicrobial stewardship program has been reported [25]. The team intervention may have an influence through items other than the initial VCM dose planning.

VCM administration was led by physicians in the non-intervention group and initial TDM was not properly performed in some cases, suggesting that some cases were excluded due to the absence of TDM data although the blood level was actually well controlled. Moreover, renal function check and blood culture re-test were basically entrusted to physician’s judgment, so that schedules of these were not specified. Accordingly, it is possible that some cases with delayed discovery of renal dysfunction due to the absence of serum creatinine measurement were included or MRSA positivity was not clarified in others despite being actually continuously positive on blood culture because the test was not performed. Blood culture test was not repeated within 30 days in 32.7% (16/49 cases) in the non-intervention group and 17.9% (5/28 cases) in the intervention group, suggesting that more actually positive patients were included in the non-intervention group.

The MIC of VCM for MRSA was not examined in detail and we could not calculate each patient’s VCM AUC due to the lack of VCM blood concentration data. It has been reported that high VCM MICs may contribute to treatment failure in patients with MRSA bacteremia [24, 32]. Moreover, VCM’s clinical effectiveness could be associated with AUC/MIC [5, 19].

Although data analysis was performed using the Cox proportional hazards model to control for confounding factors, adjustment with current/previous healthcare and medication utilization, previous infections, source of infection, comorbid infections, other antibiotics used, ICU level care, sepsis, specific comorbidities, or time was not performed. Therefore, confounding factors may have been present.

A multi-centered study with large sample sizes is required to clarify the relationship between VCM treatment failure and initial VCM dose planning by pharmacist interventions.

## Conclusions

This is the first report of VCM treatment failure based on the composite endpoint of four factors showing a significant difference between pharmacist intervention and non-intervention groups.
